# The Analysis of Variants in the General Population Reveals That *PMM2* Is Extremely Tolerant to Missense Mutations and That Diagnosis of PMM2-CDG Can Benefit from the Identification of Modifiers

**DOI:** 10.3390/ijms19082218

**Published:** 2018-07-30

**Authors:** Valentina Citro, Chiara Cimmaruta, Maria Monticelli, Guglielmo Riccio, Bruno Hay Mele, Maria Vittoria Cubellis, Giuseppina Andreotti

**Affiliations:** 1 Dipartimento di Biologia, Università Federico II, 80126 Napoli, Italy; vale.ctr@gmail.com (V.C.); chiaracimmaruta@yahoo.it (C.C.); maria.monticelli@yahoo.com (M.M.); gugli.riccio@libero.it (G.R.); arfalas@gmail.com (B.H.M.); 2 Dipartimento di Scienze Agrarie ed Agroalimentari, Università Federico II, 80055 Napoli, Italy; 3 Istituto di Chimica Biomolecolare—CNR, 80078 Pozzuoli, Italy; gandreotti@icb.cnr.it

**Keywords:** disorder of glycosylation, variant analysis, clinical informatics, modifier genes

## Abstract

Type I disorders of glycosylation (CDG), the most frequent of which is phosphomannomutase 2 (PMM2-CDG), are a group of diseases causing the incomplete *N*-glycosylation of proteins. PMM2-CDG is an autosomal recessive disease with a large phenotypic spectrum, and is associated with mutations in the *PMM2* gene. The biochemical analysis of mutants does not allow a precise genotype–phenotype correlation for PMM2-CDG. *PMM2* is very tolerant to missense and loss of function mutations, suggesting that a partial deficiency of activity might be beneficial under certain circumstances. The patient phenotype might be influenced by variants in other genes associated with the type I disorders of glycosylation in the general population.

## 1. Introduction

PMM2-CDG is the most common disorder of glycosylation (CDG) and is caused by mutations in the *PMM2* gene, impairing the activity of phosphomannomutase 2 (PMM2). CDGs affecting *N*-glycosylation can be subdivided into two groups, type I defects that involve synthesis and transfer of lipid-linked oligosaccharides (LLOs) and type II defects that impair the modification process of protein-bound oligosaccharides. PMM2-CDG, also known as CDG-Ia (or Jaeken syndrome), belongs to the first group.

The first step towards protein *N*-glycosylation requires the interconversion of mannose 6-phosphate (M6P) into mannose 1-phosphate (M1P). In humans, there are two paralogous enzymes, PMM1 and PMM2 [1], both requiring glucose 1,6 bisphosphate (G16) or mannose 1,6 bisphosphate (M16) as an activator. Both enzymes are mutases, but PMM1 has phosphatase activity too (i.e., it is able to hydrolyze G16). The role of PMM1 might be critical, because it can either support PMM2 by contributing to M6P isomerization or counteract PMM2 by hydrolyzing the activator G16 [2,3]. M1P is a precursor to GDP-mannose, which is necessary for the synthesis of dolichol phosphate mannose and lipid-linked oligosaccharide (LLO). The synthesis of LLO requires the activity of several enzymes in a multistep process that results in the addition of two *N*-acetyl glucosamines, nine mannoses, and three glucoses. The oligosaccharyltransferase, which transfers the oligosaccharide from LLO to the accepting protein, has a reduced affinity for immature LLOs [4] and, as a consequence, the *N*-glycosylated proteins are less abundant, when the pathway leading to the LLO synthesis is defective.

CDG-I can be diagnosed by the identification of hypoglycosylated liver-derived serum glycoproteins and liver proteins [5,6], in particular, transferrin [4]. However, as this test is not specific, a molecular diagnosis of PMM2-CDG through the *PMM2* gene sequencing is needed. More than 110 pathological mutations have been associated with PMM2-CDG. Usually, patients have compound heterozygotes with one inactivating mutation, Arg141His being by far the most common allele [7,8], and one hypomorphic mutation. More rarely, they carry two different hypomorphic mutations in heterozygosity or one hypomorphic mutation in homozygosity. Correlating the phenotype to genotype is made difficult by the existence of a large number of mutations and a relatively small number of patients [9]. The study of the properties of single *PMM2* mutants [10,11,12,13] or, in rare cases, of heterodimeric *PMM2* mutants [14], after their expression in *E. coli* or in human fibroblasts, has been used to determine their biochemical phenotype and to attempt a correlation with the clinical phenotype. It is worth remembering that the enzyme is an obligate dimer and studying activity/stability of single mutant homodimers is a shortcoming of the analysis [14]. Here, we describe the study of the mutants Val129Met and Val231Met and compare the properties of the purified proteins with the phenotype of the patients to illustrate the limitation of this method. We show that *PMM2* is very tolerant to missense mutations. The lack of correlation between the biochemical and clinical phenotypes might be explained, in part, by considering that the partial loss of PMM2 activity is only one of the determinants, and that other gene variants contribute to the severity of PMM2-CDG. We propose that other partially deficient genes act as modifiers of PMM2-CDG and present a list of candidate variants.

## 2. Results

### 2.1. Clinical Phenotypes and “Biochemical” Phenotypes of Phosphomannomutase 2 (PMM2) Mutants Do Not Correlate

To predict the severity of PMM2-CDG, one could measure the stability and residual activity of the mutant proteins expressed in *E. coli* or in eukaryotic cells [10,11,12,13,14]. We followed this approach for two mutants that present the same conservative substitution, Val to Met. Val129Met and Val231Met are among the most frequent mutations and have been encountered in several countries. The genotypes Val129Met/Arg141His and Val231Met/Arg141His including these mutant alleles have been associated with severe phenotypes in unrelated families, in seven and eighteen reported cases, respectively [11,15,16,17,18,19].

We measured not only the catalytic parameters, but also the long-term stability and melting temperature of these mutant proteins by thermal shift assay (Table 1), as it cannot be excluded that the role played by PMM2 in the cell does not require enzymatic activity. The melting temperatures are related to the thermodynamic stability of proteins [20], whereas the long-term measurements consent to compare the stability of different mutations at the same temperature.

For comparison, we added the data for wild-type (wt)-PMM2 and Phe119Leu, which are well-characterized pathological mutations [22]. Val129Met retains more than 50% activity under our experimental conditions and is slightly less stable than wild-type, whereas Val231Met has residual activity and its stability is lower than that of a well-founded severe mutation, Phe119Leu [22]. Our data on the relative activity and stability of Val129Met and Val231Met are in line with those reported by van Schaftingen and collaborators [10]. 

**Figure 1:**
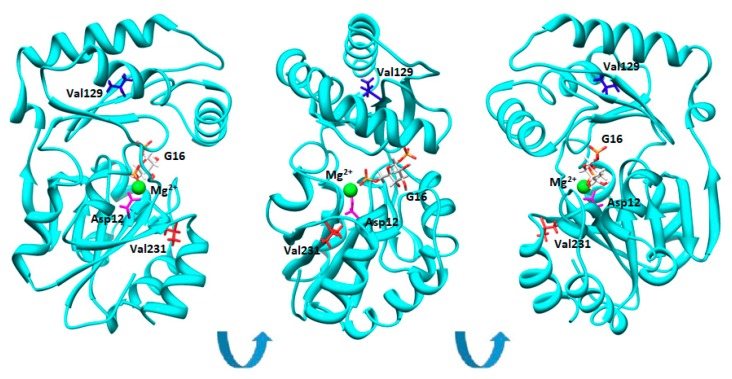
Val129 and Val231 in the structure of phosphomannomutase2. Phosphomannomutase 2 (PMM2) (single chain) is represented as cartoons. Asp12, Val129, and Val231 are shown as sticks in magenta, blue, or red, respectively. Glucose-1,6-bisphosphate (G16) is represented by sticks and is colored by atom types. Mg^2+^ is represented by a green sphere. Different orientations of the same molecule are shown.

In Figure 1, we show the model of PMM2 in the closed conformation induced by G16 binding [21].

Neither Val129 nor Val231 are in contact with the ligand and hence they do not belong to the active site. Yet, Val231 is closer than Val129 to the catalytic center of PMM2, Asp12, which is the nucleofile transferring the phosphate from the O6 to O1 of mannose. Val231 is invariant in the PMM2 as well as in PMM1 ortologues, while Val129M is not conserved [23].

Therefore, although the two mutations affect the PMM2 activity in a very different manner, the phenotype of the patients with the Arg141His/Val129Met or Arg141His/Val231Met genotypes is similarly severe.

### 2.2. Missense and Loss of Function Mutations in PMM2 Are Unexpectedly Frequent in the General Population

With a final data set spanning more than 60,000 individuals, the Exome Aggregation Consortium (ExAC) provides a unique possibility for evaluating the occurrence of *PMM2* variants in the general population [24]. The team of ExAC provided the number of observed and expected rare (MAF < 0.1%) variants per gene and separated them into synonymous, missense, and loss of function mutations (stop-gained and essential splice sites) [24]. From the original file we extracted the genes that were annotated in UniProt with the keyword “disease” [24]. From this set we subtracted the genes that are associated with autosomal dominant diseases [25], obtaining 3125 instances on which the analysis was carried out.

**Figure 2:**
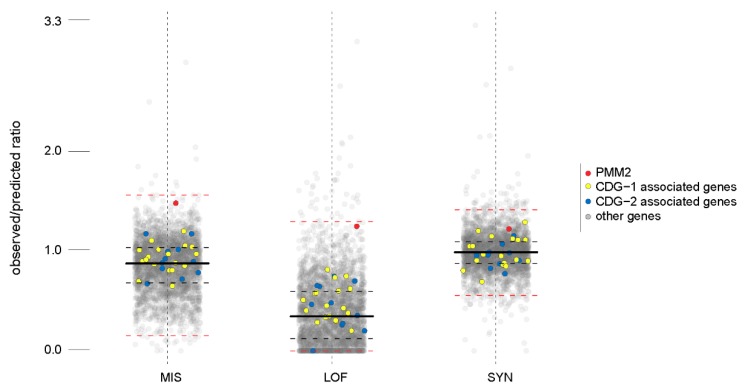
Observed versus expected variants for disease genes. Box-plots are drawn for the ratios between the number of observed and predicted variants in the general population for disease genes. Each dot represents a gene, the red dot is PMM2, yellow dots are the other genes associated with glycosylation (CDG)-I, and the blue dots are the genes associated with CDG-II. The medians are indicated by thick black lines; the lower and upper quartiles, representing observations outside the 25–75 percentile ranges, are indicated by dashed black lines; and the relative minimum and relative maximum values are indicated by dashed red lines.

We observed that the ratio between the number of observed and predicted missense mutations for *PMM2* is abnormally high for a gene associated with a human disease, even excluding the genes with autosomal dominant inheritance (Figure 2).

As can be noticed in Figure 2, a high ratio of observed versus predicted missense mutations is peculiar for *PMM2* and is not a common feature among the CDG associated genes. The ratio of observed versus predicted loss of function mutations, for *PMM2*, is above the median as well.

In supplementary File S1, we provide the list of non-singleton missense mutations in the *PMM2* extracted from ExAC [26] with the occurrence of the mutation in the active site and reference in HGMD (when available) [27]. In supplementary File S2, we provide annotations concerning the deleteriousness of the mutations obtained running 12 different predictors. We run PolyPhen2 [28], SIFT [29], LRT [30], MutationAssessor [31], PROVEAN [32], metaSVM [33], metaLR [33], MutationTaster [34], FATHMM [35], and fathmm-MKL coding [36], which provide a discrete classification of variants (classifying predictors) as well as CADD [37], which provides raw scores and scaled scores (CADD phred score). CADD is not limited to missense variants, and takes into account not only the effect on the protein, but also other features such as the proximity of the nucleotide change to an exon-intron junction, and so on. The outputs of the classifying predictors differ; SIFT and PolyPhen2 appear to have the least tendency to overestimation and provide the smallest ratio of deleterious mutation (supplementary File S2).

**Figure 3:**
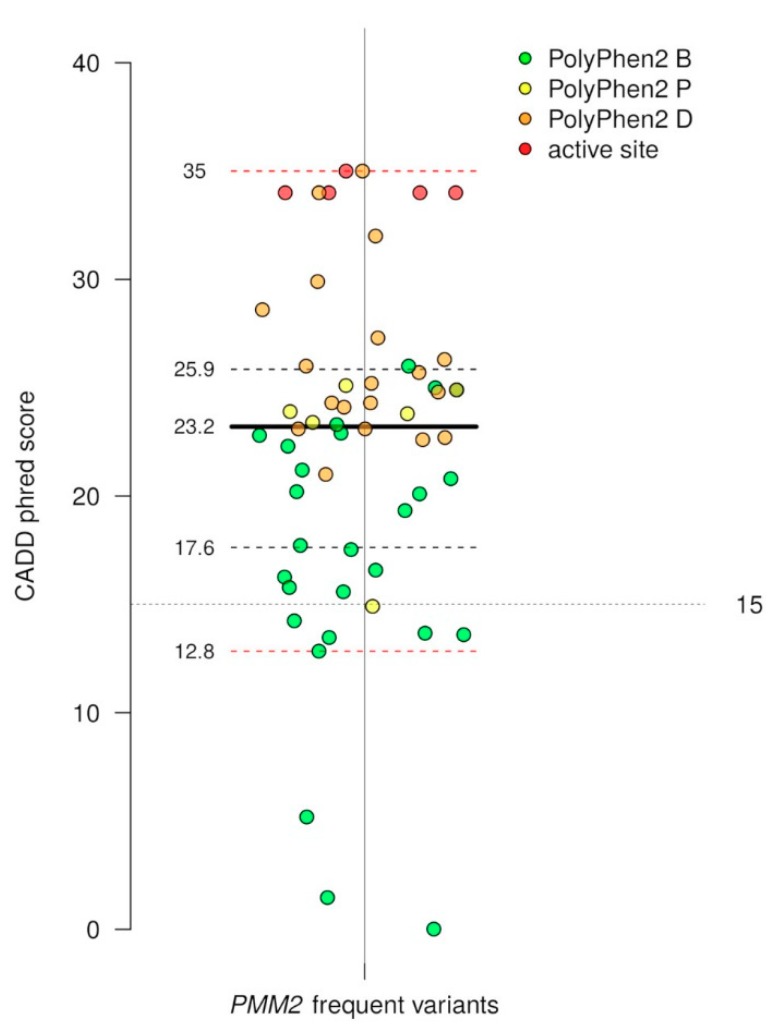
Distribution of CADD scaled scores and PolyPhen2 deleteriousness predictions for PMM2 frequent variants. The medians are indicated by thick black lines; the lower and upper quartiles, representing observations outside the 25–75 percentile ranges, are indicated by dashed black lines; and the relative minimum and relative maximum values are indicated by dashed red lines.

Excluding singletons, which are mutations observed in a single person, 56 missense mutations were observed, 50% of which are predicted as deleterious according to PolyPhen2 [28], SIFT [29], and CADD [37]. No loss of function or missense mutation that is predicted as deleterious by PolyPhen2 has ever been observed in homozygosity in the general population. The CADD scaled scores (phred score) for the *PMM2* non-singleton variants observed in the general population, are reported in Figure 3. Each dot represents a variant, the green dots are the variants that are predicted as benign by PolyPhen2, the orange dots are those predicted as probably damaging by PolyPhen2, the yellow dots are possibly damaging by PolyPhen2, and the red dots are those occurring in the active site. Although the CADD does not classify variants, it was suggested that 15 be used as a cut-off value to distinguish the deleterious missense mutations. The CADD and Polyphen2 agree substantially, and the raw scores correlate with an *r* value of 0.80 and a *p* value < 0.0001 (data in supplementary File S2).

Missense mutations have been observed in *PMM2* with a relatively low frequency, excluding Glu197Ala, which was found in approximately 2% of the general population in all ethnic groups, with the exception of Eastern Asians (supplementary File S1). This variant has been observed in association with Arg141His in patients with the moderate phenotype [17,38]. The effect of this mutation on the protein expressed in *E. coli* is mild [11,39].

### 2.3. Genes Involved in Congenital Disorders of Glycosylation I (CDG-I) Pathologies Have Frequent Variants in the General Population

The severity of PMM2-CDG could depend on the genetic background. Indeed this hypothesis could explain many reports concerning the lack of correlation between the genotype and phenotype [9,40,41]. Mutations affecting the other genes involved in the type I CDGs could act as modifiers of the PMM2-CDG phenotype. We extracted these mutations from ExAC and found that many of them are relatively frequent and some of them are predicted as deleterious by PolyPhen2, which is the tool most commonly used for variant annotation, as well as by other predictors, such as SIFT [29], LRT [30], MutationAssessor [31], PROVEAN [32], metaSVM [33], metaLR [33], MutationTaster [34], FATHMM [35], fathmm-MKL coding [36], and CADD [37]. In supplementary File S2, we provide the complete list of deleteriousness predictions, and in supplementary File S3, we provide all of the data extracted from ExAC for the frequent mutations in type I CDG genes (allelic frequency >0.001) and the reference in HGMD (when available) [27]. In Table 2, we provide the list of potentially damaging mutations according to PolyPhen2 and CADD, with the accordance obtained with eleven different classifiers (number of non-benign prediction) and with their allelic frequency. Altogether, the allelic frequency of deleterious mutations in the CDG-I genes, excluding *PMM2*, is above 0.30. The most frequent deleterious variants are Ala229Thr in *MPDU1*, Ile393Val in *ALG12*, and Arg268Gln in *ALG8* (Table 2). All of these variants are panethnically distributed (supplementary File S3).

## 3. Discussion

A number of missense mutations higher than that expected for a gene associated with a recessive disease is observed in PMM2. Half of them are predicted as deleterious. This finding experimentally supports the hypothesis that has been put forward by Freeze [42], that many different mutations have arisen in *PMM2* and have not been negatively selected, possibly because a reduced mannomutase activity has a beneficial role under certain conditions. For other genes with a high ratio between the observed and predicted missense mutation, a protective effect has been reported. A few examples will be cited, namely: CD36, which is responsible for Platelet glycoprotein IV deficiency, but is protective against atherosclerosis [43]; MTUS1, which associated to Hepatocellular carcinoma, but plays a protective role against inflammation [44]; and DAOA, which is associated with Schizophrenia, but is related to better cognitive performance [45].

People carrying one wild-type *PMM2* allele and one inactivating mutation on the other allele are asymptomatic. Hence, it is accepted that a 50% PMM2 activity should be sufficient [46]. When both alleles carry a mutation, the residual activity can drop below the safety threshold, and the symptoms of the PMM2-CDG can be manifested in a severe, moderate, or mild form.

Predicting the severity of the disease is difficult, even when the mutation present in each allele is known. A thorough biochemical characterization of the purified *PMM2* mutants can possibly distinguish between the extreme ends of the phenotype, but the genetic background should be taken into account for the grading diagnosis. Actually, it has been observed that the presence of the variant Phe304Ser in the gene *ALG6* can worsen the PMM2-CDG phenotype [47,48]. Phe304Ser_ALG6 was also found in the homozygous state in a severe CDG-I associated with dehydrodolichol diphosphate synthase deficiency [49]. Phe304Ser_ALG6 is indeed one the most frequent variants observed in the general population, both in heterozygosity and homozygosity [26]. Although it is not a disease variant [50], Phe304Ser_ALG6 is less fit than the wild-type in restoring the glycosylation of glycoprotein carboxypeptidase Y when introduced into a yeast strain deleted for *ALG6*. We showed that Phe304Ser_ALG6 is not the only candidate modifier. In fact, the data reported in ExAC for the genes associated with CDG-I reveal that other missense mutations are relatively frequent in the general population, some of which are probably deleterious. We do not yet know if these variants can modulate PMM2-CDG, but this is certainly something that should be assessed in patients.

## 4. Materials and Methods

DEAE-Sepharose ff and Superdex-75 were purchased from GE Healthcare Life Sciences, Milan, Italy. Phosphoglucose isomerase from rabbit muscle, phosphomannose isomerase from *E. coli*, glucose 6-phosphate dehydrogenase from baker’s yeast (*S. cerevisiae*), phosphoglucomutase from rabbit muscle, α-d(+)Mannose 1-phosphate sodium salt hydrate, and β-Nicotinamide adenine dinucleotide phosphate sodium salt, were purchased from Sigma-Aldrich, Milan, Italy. Sypro Orange was from Invitrogen Molecular Probes, Monza, Italy. Mannose-1,6-bisphosphate was synthesized and purified, as described [3]. All of the other reagents were of analytical grade.

### 4.1. Protein Expression and Purification

Val129Met-PMM2 and Val231Met-PMM2 were expressed in *E. coli* (BL21[DE3] strain, using the Pet22b+ expression vector) and purified following a protocol similar to that applied for the wild-type PMM2 [13,14,51]. The bacteria were grown at 37 °C in an LB broth in the presence of ampicillin 0.2 mg/mL and isopropyl 1-thio-β-d-galactopyranoside; 0.4 mM was added when the optical density was 0.5 in order to induce the expression of the proteins. The culture was prolonged for 4 h, then the cells were harvested; washed with PBS; suspended in Tris 50 mM, pH 7.5 containing 2-mercaptoethanol 1 mM, EDTA 5 mM, and phenylmethylsulfonyl fluoride 1 mM; and lysed with lysozyme 1 mg/mL. MgCl_2_ 10 mM was added before adding Deoxyribonuclease I 0.005 mg/mL. The clear homogenate was recovered and a salting-out step was realized by adding ammonium sulfate to a 60% saturation. The recovered material was dissolved and dialyzed (in Hepes 50 mM, pH 7.1 containing 2-mercaptoethanol 1 mM), then loaded onto a DEAE-Sepharose Fast Flow column equilibrated with the same buffer. The pass-through was collected. A final fractionation step on a Superdex 75 column (equilibrated in Hepes 20 mM, MgCl_2_ 1 mM, NaCl 150 mM, pH 7.5) was conducted. The active fractions judged pure by SDS-PAGE were pooled and concentrated.

### 4.2. Enzymatic Assays

Human phosphomannomutases catalysed the conversion of glucose-1 phosphate (G1P) into glucose 6-phosphate (G6P), as well as that of M1P into M6P [3,51].

The phosphomannomutase activity (i.e., isomerization of M1P in the presence of M16) was measured spectrophometrically at 340 nm, at 32 °C, in Hepes 20 mM, pH 7.5 containing MgCl_2_ 1 mM, NaCl 150 mM, NADP^+^ 0.25 mM, BSA 0.1 mg/mL, glucose-6-phosphate dehydrogenase 0.01 mg/mL, phosphoglucose isomerase, 0.01 mg/mL, and phosphomannose isomerase 0.0032 mg/mL. In order to measure the kinetic parameters, the concentration of M1P was varied from 0 to 600 μM, while the M16 was held constant at 3 μM, or the concentration of M16 was varied from 0 to 60 μM, while M1P was kept constant at 200 μM.

The phosphoglucomutase activity was measured similarly using Hepes 20 mM, pH 7.5 containing MgCl_2_ 1 mM, NaCl 150 mM, NADP^+^ 0.25 mM, BSA 0.1 mg/mL, glucose-6-phosphate dehydrogenase 0.01 mg/mL, G1P 100 μM, and G16 100 μM as the activator.

### 4.3. Thermal Stability

The melting temperatures of thepure proteins (0.6 mg/mL) were measured by thermal shift assay conducted with the StepOne Real-Time PCR System (Applied Biosystems, Foster City, CA, USA), as already described [52]. Briefly, the experiment was conducted in Hepes 20 mM pH 7.5, containing MgCl_2_ 1 mM, NaCl 150 mM, dithiothreitol 1 mM, and Sypro Orange 2.4×, and the samples were heated from 20 to 90° at 1 °C/min, with increments of 0.6 °C.

The long-term stability of wt-PMM2, Val129Met-PMM2, and V231M-PMM2 was monitored at 40 °C. The pure proteins (0.03 mg/mL in Hepes 20 mM pH 7.5, containing MgCl_2_ 1 mM, NaCl 150 mM, and BSA 0.1 mg/mL) were incubated and the aliquots were taken out at suitable time intervals, cooled on ice, and the residual phosphoglucomutase activity was measured. Another experiment was conducted in parallel, in the presence of G16 200 μM.

### 4.4. Miscellaneous

The protein concentrations were routinely estimated using the Quick Start Bradford (Bio-Rad, Hercules, CA, USA), with BSA as the standard [53]. SDS-PAGE was performed using standard procedures [54].

The data concerning the number of observed and predicted synonymous, missense, and loss of function mutations were extracted from the supplementary Table 13 in the literature [24]. The disease genes are those annotated in UniProt with the keyword “disease”. The genes associated with the diseases with a dominant inheritance were obtained from the supplementary File S2 in the literature [25]. The disease genes present in supplementary Table 13 in the literature [24], minus those with dominant inheritance, constitute the set that is the object of the analysis (3125 genes).

To explore the distribution of the ratios between the observed and predicted cases for the three mutation types (MIS—missense, LOF—loss of function, and SYN—synonymous), we calculated a five-points distribution range (the lowest datum still within 1.5 IQR of the lower quartile, first quartile, median, second quartile, and the highest datum still within 1.5 IQR of the upper quartile) using the function boxplot() from the R graphics package [55]. We then plotted the observed/predicted ratio for each one of the 3125 genes contained in the file, with respect to the mutation class (MIS, LOF, and SYN), scattering them using a random x-jitter (±2) to improve the information display. We associated the specific colors to PMM2 (red), CDG1-associated genes (yellow), and CDG2-associated genes (blue). On top of the point-based visualization, we overlaid the five-point distribution range.

The data concerning the allelic frequencies in the CDG-I genes were extracted from the literature [26], excluding variants whose consequences are in a non-canonical transcript.

The active site residues were identified with DrosteP [56] on the model PMM2 in closed conformation [21]. The figure describing the structure of PMM2 and the location of Val129 and Val231 was prepared with CHIMERA [57].

The deleteriousness predictions of PolyPhen2 [28], SIFT [29], LRT [30], MutationAssessor [31], PROVEAN [32], metaSVM [33], metaLR [33], MutationTaster [34], FATHMM [35], fathmm-MKL coding [36], and CADD [37] were obtained running wANNOVAR [58]. The details about the categorical prediction can be found in ANNOVAR users’ guide [59].

## Figures and Tables

**Table 1:** Biochemical phenotype of some phosphomannomutase 2 (PMM2) variants. M1P: mannose 1-phosphate.

PMM2	Relative Activity	Km M1P (µM)	Experimental Conditions for Km M1P	Km M16 (µM)	Experimental Conditions for Km M16	Long-Term Stability Residual Activity after 10 min @ 40 °C	Melting Temperature (°C)	Ref.
Wild-type	100	18	10 µM M16	1.1	200 µM M1P	100	-	[10]
	100	16.0 ± 1.5	3 µM M16	5.4 ± 0.6 *	* 200 µM M1P	100 ** 100 (in the presence of 200 µM G16) **	53.5 ± 0.9 ***	[13] * [3] ** this paper *** [21]
Phe119Leu	24.5	45	50 µM M16	4.9	200 µM M1P	~60	-	[10]
	29	11.8 ± 1.0	3 µM M16	nd	nd	~95 (37 °C)	45.0 ± 0.8 ***	[13] *** [21]
Val29Met	50	21	10 µM M16	1.1	200 µM M1P	~55	-	[10]
	89	33.0 ± 3.9	3 µM M16	3.7 ± 0.5	200 µM M1P	83 100 (in the presence of 200 µM G16)	48.1 ± 1.0	this paper
Val231Met	38.5	18	10 µM M16	1.1	200 µM M1P	~20	-	[10]
	20	6.9 ± 1.1	3 µM M16	5.9 ± 1.1	200 µM M1P	23 76 (in the presence of 200 µM G16)	40.0 ± 3.0	this paper

nd: not determined; *, **, *** indicate the source of the data.

**Table 2:** Frequent deleterious variants in type I disorders of glycosylation other than PMM2-glycosylation (CDG).

Gene	Mutation	Accordance Among Classifiers	CADD Scaled Scores	Allele Frequency
*ALG6*	p.Leu453Val	10/11	23.6	0.012
*ALG3*	p.Val362Ile	7/11	25.5	0.001
*MPDU1*	p.Ala229Thr	3/11	20.9	0.154
	p.Gly225Ser	4/11	24.1	0.010
*ALG12*	p.Ile393Val	7/11	23.4	0.112
*ALG8*	p.Arg268Gln	11/11	35	0.014
*ALG2*	p.Pro56Leu	10/11	24.2	0.001
*ALG1*	p.Thr64Asn	7/11	24.7	0.003
	p.Ala3Asp	3/11	22.6	0.001
*ALG9*	p.Ser255Leu	5/11	23.4	0.003
*RFT1*	p.Ala185Thr	8/11	26.8	0.017
	p.Ser16Cys	10/11	23.9	0.001
*SRD5A3*	p.His309Asp	7/11	15.91	0.004
*DDOST*	p.Arg315Gln	6/11	29.9	0.003
	p.Thr400Ile	10/11	32	0.001

## Supplementary Materials

Supplementary materials can be found at http://www.mdpi.com/1422-0067/19/8/2218/s1

## Abbreviations

M1P	mannose-1phosphate
M16	mannose-1,6-bisphosphate
G1P	glucose-1phosphate
G16	glucose-1,6-bisphosphate
